# Primary Investigation on the Synergistic Effects of Methyl Bromide and 1 °C Cold Treatment for Two-Spotted Spider Mite (*Tetranychus urticae*) and the Citrus Mealybug (*Planococcus citri*)

**DOI:** 10.3390/insects16040377

**Published:** 2025-04-02

**Authors:** Jin-Sung Yoo, Jae-Ho Ban, Ji-Eun Choi, Bong-Su Kim, Jun-Ran Kim

**Affiliations:** Department of Plant Quarantine, Animal and Plant Quarantine Agency (APQA), Gimcheon 39660, Republic of Korea; filton96@gmail.com (J.-S.Y.); banjh48@gmail.com (J.-H.B.); cje1993@naver.com (J.-E.C.); bskim79@korea.kr (B.-S.K.)

**Keywords:** methyl bromide, cold treatment, *Tetranychus urticae*, *Planococcus citri*

## Abstract

Since the use of methyl bromide has been prohibited globally because of environmental concerns, several alternative fumigants have been newly developed and applied to fresh products. However, single treatment with a methyl bromide alternative fumigant cannot completely replace methyl bromide treatment for some products because of issues associated with long treatment times and phytotoxicity. In this study, we compared the mortality of two agricultural pests, *Tetranuchus urticae* and *Planococcus citri*, after single treatment with methyl bromide and combined application of methyl bromide and cold treatment to confirm the synergistic effects of chemical and physical treatments. The combined application of methyl bromide and cold treatment was effective against the nymph and adult stages of *T. urticae*, but no synergism was observed at the egg stage. For *P. citri*, the required dosage of methyl bromide decreased when methyl bromide treatment was followed by low temperature, possibly because of the susceptibility of *P. citri* to cold treatment. These results indicate that the synergism of fumigants with cold treatment can differ by pest species and growth stage, and further studies on other pests are needed to reduce methyl bromide usage.

## Simple Summary

Methyl bromide has been designated as an ozone depleting substance under the Montreal Protocol since 1987; however, single treatment with methyl bromide has long been used in plant quarantine, and some products still require methyl bromide during plant quarantine. In this study, we focused on reducing the methyl bromide dosage by developing a combined treatment method of methyl bromide and cold treatment. *Tetranychus urticae* was more tolerant of low temperature than *Planococcus citri*, and the egg stage of *T. urticae* was the least tolerant of both methyl bromide and cold treatment. Compared with *T. urticae*, *P. citri* was more tolerant of methyl bromide, but all growth stages of *P. citri* showed very weak tolerance to cold treatment. The synergistic effect ratios for nymphs and adults of *T. urticae* were 1.4 and 2.6, respectively, while *P. citri* showed higher values. These results demonstrate the potential for combined application in the control of pests. The combination of methyl bromide and cold treatment is expected to be applicable to various pests and crops, and further studies on other pests are needed to reduce methyl bromide usage.

## 1. Introduction

In plant quarantine, single-fumigant treatment is a commonly used method to eradicate invasive pests. Single treatment with methyl bromide exhibits broad-spectrum efficacy against quarantine pests within short exposure periods of less than 48 h [1]. As the use of methyl bromide has been prohibited due to environmental concerns, alternative treatments have been developed, and many of these alternatives are fumigants [2,3]. Ethyl formate, a methyl bromide alternative fumigant for fruits and vegetables, has rapid insecticidal effects on fresh products and phosphine, a methyl bromide alternative fumigant for grains and nursery plants, also has the potential to control pests and exhibits low phytotoxicity [4,5]. Methyl bromide alternative fumigants have already been commercialized and are used as single treatments in several countries [6,7]. However, single treatment with fumigants can be problematic for some fresh products. Phosphine requires a long fumigation period, normally more than 24 h, to kill insects, and a high dosage of ethyl formate can lead to economic loss for some fresh products [8,9].

Physical treatment methods, such as cold treatment or heat treatment, have long been in use. Heat treatment has shown good efficacy against grain pests, and cold treatment can control insects with relatively little damage to products [3]. However, the application of physical treatment alone can be performed for only a few products because of the long exposure time (for cold) or possible damage to fresh products (heat). Combined application of fumigants and physical treatment to enhance efficacy and decrease phytotoxicity has recently been reported. Haritos et al. [10] reported enhancement of the efficacy of ethyl formate when combined with carbon dioxide. Jamieson et al. [11] reported a decrease in the treatment time for ethyl formate under low-oxygen conditions. Kwon et al. [12] reported that combined treatment with ethyl formate and low temperature was effective in controlling *Drosophila suzukii*. These results indicate that combined application of fumigants and physical treatment is a potential method for controlling quarantine pests.

Combined application of methyl bromide with cold treatment has long been used to control lepidopterous pests and fruit flies [13,14,15]. In our previous research [16], we reported that compared with single treatment with methyl bromide, combined application of methyl bromide and cold treatment enhanced the control efficacy against *D. suzukii*, an important quarantine pest in Korea. Lee et al. [17,18] performed metabolic profiling in *D. suzukii* after combined treatment with fumigants, namely ethyl formate and phosphine, and cold. In this study, we assessed the synergistic effects of the fumigant methyl bromide and cold treatment on two agricultural pests, the citrus mealybug (*Planococcus citri* (Risso)) and two-spotted spider mites (*Tetranychus urticae* (Koch)), as *P. citri* is highly tolerant of fumigants and *T. urticae* is highly tolerant of low temperatures [19,20].

## 2. Materials and Methods

### 2.1. Tested Insects

*P. citri* and *T. urticae* were reared at the Plant Quarantine Technology Center in South Korea. Both species were maintained in acrylic cages (W 30 × D 30 × H 60 cm; MegaView Science Co., Ltd., Taichung City, Taiwan) at 25 ± 1 °C under 60 ± 10% relative humidity (RH) with a 16:8 h light:dark photoperiod. *T. urticae* was reared on bell pepper plants grown in the greenhouse of APQA, and *P*. *citri* was reared on potatoes purchased from a traditional market. The replacement cycles for food plants for *T. urticae* and *P. citri* were 2 and 4 weeks, respectively. Both food plants were placed on a stainless-steel tray (W 530 × D 430 × H 80 mm), and a sticky trap was placed around the tray to prevent contamination and insect escape. The developmental stages that were used for these experiments were the egg, larva, pupa, and adult stages, and insects at each stage were moved to breeding dishes for test using a soft brush. Approximately 30 to 40 test insects were used per replication, and at least three replications were tested for statistical analysis.

### 2.2. Fumigant and Fumigation Methods

Methyl bromide (98.5%) was purchased from Nonghyup Chemicals, Seongnam-si, Korea. Methyl bromide gas sampled from the methyl bromide cylinder was added to a 10 L Tedlar bag (SKC Inc., Eighty Four, PA, USA) to prepare for the test. Afterward, a certain amount of gas was extracted from the 10 L Tedlar bag, injected into a 55 L desiccator (UBNC, Incheon, Republic of Korea) and well-sealed. The desiccator was equipped with a 100 mm diameter fan (Uni BNC, Goyang, Republic of Korea) to circulate the air and fumigant, and a HOBO (model H-08-004-02, Onset Computer Corporation, Bourne, MA, USA) data logger was placed at the bottom of the desiccator to measure the temperature and humidity inside. Methyl bromide treatment was carried out at concentrations of 2, 5, 10, 15, 20, 30, and 40 g/m^3^ for *T. urticae* and 5, 10, 15, 20, 25, 30, 40, and 50 g/m^3^ for *P. citri*.

### 2.3. Measurement of Fumigant Concentration

After the methyl bromide gas was injected into the desiccator, the gas concentration inside the desiccator was measured hourly. The mixed gas in the desiccator was sampled via a 100 mL syringe, and the collected gas was injected into a 1 L Tedlar bag and stored until gas chromatography (GC) analysis. To perform concentration analysis, gas was sampled via a 100 μL gas-tight syringe (SGE Analytical, Australia) and then injected into a GC instrument (7890A, Agilent Technologies, Santa Clara, CA, USA). The concentration of the gas was calculated on the basis of peak height and area compared with those of external standards. The GC conditions for these measurements were as follows: Rtx-5 column (15 m × 250 μm × 1 μm; Restek, Bellefonte, PA, USA), split mode (10:1), flame ionization detector (FID), air flow (300 mL/min), H_2_ flow (30 mL/min), make-up gas (N_2_) injector temperature (250 °C), oven temperature (200 °C), detector temperature (250 °C), and injection volume (70 μL).

### 2.4. Cold Treatment (1 °C)

The desiccator was placed in a 20 ft reefer container (W 235 × L 590 × H 239 cm), and the temperature of the container was lowered to perform cold treatment. A small circulating fan was placed at the bottom of the desiccator, and the breeding dishes with test insects were placed on a slotted plastic plate above the fan. The temperature of the container was set to the experimental setting of 1.0 °C before the experiment. The temperature and humidity were measured via a HOBO data logger until the end of the experiment. Since the experiment for the spotted spider mites was conducted for 1 to 10 days, five desiccators were used for each experiment. After the scheduled date, each desiccator was opened, and insect mortality was calculated. For *P. citri*, cold treatment was performed for 4, 8, 24, and 48 h, and 4 desiccators were used per experiment. The mortality rate was evaluated by touching the pest with a soft brush; the pest was determined to be dead if it did not move. The experiments were conducted with three biological replicates.

### 2.5. Determination of the Concentration × Time (Ct) Product

The relationship between the concentration of a fumigant and time can be described by Equation (1), as described by Daglish et al. [20]. After fumigation, the concentration in the desiccator decreases over time, so Equation (1) can be used to calculate how much fumigant or time is required to control insects.

(1) $$C^{n}T=k,$$

where C = the concentration, n = the constant that determines the rate of decrease in concentration, T = time, and k = a specific constant.

To calculate the Ct product, the concentrations of methyl bromide used were 2, 5, 10, 15, 20, 30, and 40 g/m^3^, and the concentration of methyl bromide used for actual fumigation treatment was calculated using Equation (2), as described by Ren et al. [21].

(2) $$\mathrm{Ct}=\sum \frac{\left(C_{i}+C_{i+1}\right)\left(t_{i+1}-t_{i}\right)}{2},$$

where C = concentration of fumigant (g/m^3^), t = time of exposure (h), i = order of measurement, and Ct = concentration × time product (g∙h/m^3^).

The dosage of the methyl bromide fumigant to be injected into the 55 L desiccator was calculated via Equation (3).

(3) $$V_{f}=(1\;-\;\frac{T}{273})(\frac{1.7\;\times \;10^{4}\times C\times V}{P\times M\times N})$$

where V_f_ = actual fumigant gas treatment volume (mL), T = temperature (°C), C = intended concentration of fumigant (g/m^3^), V = volume of fumigation desiccator (L), P = pressure (mm Hg), M = molecular weight of fumigant, and N = purity of gas (%).

### 2.6. Statistical Analysis

The lethal concentration time 50% (LCt_50_) and 99% (LCt_99_) values for methyl bromide against *P. citri* and *T. urticae* were analyzed using the probit analysis program. This program analyzes the quantitative response data on the basis of Finney’s method [22]. The means for all treatment groups were separated by ANOVA. To reduce the probability of making an error in rejecting the null hypothesis, the alpha level was fixed at 0.05.

## 3. Results

### 3.1. Efficacy of Treatment with Methyl Bromide Alone on T. urticae and P. citri

When *T. urticae* was treated with methyl bromide, complete control of the egg stage was observed at a concentration of 10 g/m^3^, while complete control of the nymph stage and adult stage required more than 30 g/m^3^ (Table 1). The LCt_50_ value of single treatment with methyl bromide for *T. urticae* was highest at the nymph stage (15.70 g∙h/m^3^) and lowest at the adult stage (6.71 g∙h/m^3^), but the LCt_99_ value of single treatment with methyl bromide for *T. urticae* was highest at the nymph stage (70.35 g∙h/m^3^) and lowest at the egg stage (17.09 g∙h/m^3^) (Table 2).

Upon treatment of *P. citri* with methyl bromide alone, all eggs, nymphs, and adults died at a concentration of 40 g/m^3^ (Table 3). The LCt_50_ and LCt_99_ values for eggs, nymphs, and adults were 16.3, 19.0, and 21.1 g∙h/m^3^ and 34.6, 97.7 and 86.4 g∙h/m^3^, respectively. The required concentration of methyl bromide was greater at the adult and nymph stages than at the egg stage (Table 4).

### 3.2. Efficacy of Cold Treatment Alone on T. urticae and P. citri

Under cold treatment alone, the LT_50_ values for the egg, nymph, and adult stages of *T. urticae* were 19.24, 36.48, and 70.31 days, respectively, which indicates that the adult stage is more tolerant of cold treatment than the egg and nymph stages (Table 5). However, the LT_99_ values were not calculated because all growth stages of *T. urticae* presented less than 50% mortality after 10 days of cold treatment.

The LT_50_ and LT_99_ values of the egg, nymph, and adult stages of *P. citri* were 8.52, 7.30, and 8.45 and 38.17, 35.36, and 28.69 h, respectively (Table 6). *P. citri* was more susceptible to cold treatment than *T. urticae*, and all growth stages of *P. citri* were more than 99% controlled within 2~3 days of cold treatment.

### 3.3. Efficacy of Combined Treatment with Methyl Bromide and Cold Treatment on T. urticae and P. citri

When methyl bromide treatment was followed by cold treatment for 1 day, 100% mortality of *T. urticae* at the egg, nymph, and adult stages was achieved at 10, 20, and 25 g/m^3^, respectively (Table 7). The LCt_50_ and LCt_99_ values for methyl bromide treatment combined with cold treatment were 9.82, 8.96, and 11.98 and 22.67, 29.47, and 45.47 g·h/m^3^, respectively (Table 8). The synergistic ratio of methyl bromide and cold treatment was high at the nymph and adult stages, but mortality at the egg stage decreased when methyl bromide treatment was followed by cold treatment (Table 9).

To control *P. citri* with methyl bromide alone, a dose of more than 40 g/m^3^ was required (Table 6), but when cold treatment was performed for 1 day after methyl bromide fumigation, 100% mortality was observed at 2 g/m^3^ for the egg stage, 10 g/m^3^ for the nymph stage, and 5 g/m^3^ for the adult stage (Table 10). Although the synergistic ratio was not calculable due to the low methyl bromide dosage, the reduction in methyl bromide dosage for complete control at all stages significantly increased. Compared with methyl bromide treatment alone, methyl bromide fumigation followed by cold treatment increased the efficacy against the egg, nymph, and adult stages by 20-, 4-, and 8-fold, respectively (Table 11).

## 4. Discussion

Several recent studies have focused on developing new fumigants as alternatives to methyl bromide. Ethyl formate and phosphine, which have been developed as methyl bromide alternative fumigants, have shown potentially useful features, such as high efficacy against pests and relatively little damage to plants. However, the applicability of these new fumigants to all crops is still limited due to problems associated with aspects such as sorption, exposure time, and phytotoxicity [6,23,24].

Single treatment with methyl bromide has long been used in plant quarantine, and some products still require methyl bromide during plant quarantine. In this study, we focused not on methyl bromide alternatives but on reducing the methyl bromide dosage. We found that single treatment of *T. urticae* and *P. citri* with methyl bromide required a high dosage for complete control at all growth stages. More than 70 g h/m^3^ was required to achieve 99% mortality of *T. urticae*, and 97 g h/m^3^ was required to achieve 99% mortality of *P. citri*. These results indicate that more than 40 to 60 g of methyl bromide should be used to control both pests as methyl bromide treatment is usually performed for 2 h.

Jalil et al. [25] reported that the efficacy of methyl bromide for mites can differ, as 19 g/m^3^ and 5 h of treatment was required at 18 °C but more than 60 g/m^3^ was required at 2 °C to control 90% of the mites. For Chilean grape, the fumigation regimen for fruit flies is 32 g/m^3^ for 3.5 h, whereas that for mealybugs is 64 g/m^3^, which can impact the degradation of grape quality [26]. These studies indicate that single treatment with methyl bromide requires a high dosage for pest control, which can affect fruit quality and the environment. In the case of cold treatment, different control effects were observed for *T. urticae* and *P. citri*. For *T. urticae*, complete control with cold treatment alone was difficult at all growth stages, and more than two weeks would be needed for complete control of *T. urticae*. However, in the case of *P. citri*, more than 99% control was observed at all growth stages within 48 h. These differences could be attributed to the cold tolerance of the insects. Dentener et al. [27] reported that mealybugs were less tolerant of cold but more tolerant of heat than moths. With respect to *T. urticae*, Lester et al. [28] reported that cold storage of the pest at 0 °C after hot water immersion did not increase the control efficacy; rather, cold storage kept the mites alive. These results indicate that the efficacy of cold treatment can differ across insect species.

Compared with single treatment, combined application of methyl bromide and cold treatment resulted in greater efficacy in both insects. In the case of *T. urticae*, combined treatment showed enhanced efficacy against the nymph and adult stages compared with treatment with methyl bromide alone. An increase in efficacy was not observed against the egg stage; in fact, the susceptibility of eggs to low temperature was greater than that of the other growth stages. In the case of *P. citri*, 100% mortality was observed at all growth stages at lower methyl bromide dosages, which indicates that cold treatment can maintain efficacy with lower methyl bromide dosage. Good synergism with cold treatment has been observed for not only methyl bromide but also other fumigants. Kwon et al. [12] reported enhanced efficacy of ethyl formate against *D. suzukii* when used in combination with cold treatment, and Jeon et al. [29] reported similar results, although some differences in synergism were observed across growth stages. Kim et al. [30] reported the synergism of phosphine and cold treatment, and combined treatment with phosphine and cold increased the control efficacy for the peach fruit moth, *Carposina sasakii*. Lee et al. [18] reported that combined treatment with ethyl formate and cold affects the biosynthesis of amino acids and nucleotides and alters the TCA cycle. In our experiment, methyl bromide also showed enhanced efficacy in both insects when combined with cold, indicating that similar metabolite changes could occur in both insect species. As the efficacy of methyl bromide against both pests increased when it was used in combination with cold, the synergistic ratios were different. The synergistic ratios for the nymph and adult stages of *T. urticae* were 1.4 and 2.6, respectively, but the synergistic ratios for the nymph and adult stages of *P. citri* was greater than those observed for *T. urticae*.

These differences are attributed to the cold tolerance of the insects. As *P. citri* is more susceptible to cold than *T. urticae*: most of the *P. citri* individuals were killed during the cold treatment period, whereas most of the *T. urticae* individuals survived. Kim et al. [16] reported that combined treatment with methyl bromide and cold can decrease the amount of methyl bromide needed to control spotted wing Drosophila, *D. suzukii*; however, it is unclear whether this increase originates from the synergistic effect of the two treatments because cold treatment itself has good efficacy against *D. suzukii*. For the same reason, the synergistic effects of methyl bromide and cold on *P. citri* are not clear, but this treatment combination can still be useful for controlling pests with a reduced amount of methyl bromide.

In our study, the combination of methyl bromide and cold treatment had a strong synergistic effect, demonstrating the potential for application in the control of plant quarantine pests. The combination of methyl bromide and cold treatment is expected to be applicable to various pests and crops; however, to apply this combined treatment in the quarantine field, further studies are required, such as a scaled-up trial to confirm the 99.9968% quarantine level mortality, changes in mortality according to different cold treatment temperatures, and phytotoxicity tests on several fruits and vegetables.

## Figures and Tables

**Table 1:** Mortality of *T. urticae* fumigated with methyl bromide.

Concentration (g/m^3^)	Egg	Nymph	Adult	Ct ^a^ Value (g∙h/m^3^)
	Mortality ± SE ^b^ (%)	Mortality ± SE (%)	Mortality ± SE (%)	
0	0.0 ± 0.0 a	0.0 ± 0.0 a	0.0 ± 0.0 a	0
2	2.8 ± 0.3 b	4.2 ± 2.2 b	4.1 ± 2.1 a	3.8
5	36.5 ± 3.4 c	19.1 ± 4.1 c	26.2 ± 4.0 b	8.2
10	100.0 ± 0.0 d	45.8 ± 3.0 d	62.9 ± 10.7 c	15.9
15	100.0 ± 0.0 d	51.1 ± 11.9 d	68.9 ± 6.9 c	23.3
20	100.0 ± 0.0 d	97.7 ± 1.1 e	89.8 ± 5.6 d	32.9
30	100.0 ± 0.0 d	100.0 ± 0.0 f	100.0 ± 0.0 e	48.8
40	100.0 ± 0.0 d	100.0 ± 0.0 f	100.0 ± 0.0 e	67.0

^a^ CT = concentration × time. ^b^ SE = standard error. Mean values within each column followed by the same letter are not significantly different (*p* < 0.05).

**Table 2:** Probit analysis of the mortality of *T. urticae* fumigated with methyl bromide.

Fumigant	Stage	*n*	LCt_50_ (g∙h/m^3^) (95% Conf. Limits)	LCt_99_ (g∙h/m^3^) (99% Conf. Limits)	Slope ± SE	*Df*	χ^2^
Methyl bromide	Egg	1910	8.55 (2.39–12.83)	17.09 (10.14–20.94)	7.74 ± 1.60	7	201.02
	Nymph	733	15.70 (9.60–21.86)	70.35 (42.54–272.92)	3.57 ± 0.70	7	1398.1
	Adult	768	13.28 (10.83–16.54)	66.95 (43.19–167.51)	3.63 ± 0.59	7	251.11

**Table 3:** Mortality of *P. citri* fumigated with methyl bromide.

Concentration (g/m^3^)	Egg	Nymph	Adult	Ct Value (g∙h/m^3^)
	Mortality ± SE (%)	Mortality ± SE (%)	Mortality ± SE (%)	
0	0.0 ± 0.0 a	0.0 ± 0.0 a	0.0 ± 0.0 a	0
5	4.5 ± 0.9 a	-	11.5 ± 4.6 a	4.5
10	28.9 ± 6.7 a	17.1 ± 4.2 b	23.3 ± 4.6 b	14.7
15	-	22.4 ± 3.6 b	-	14.6
20	89.7 ± 1.4 b	50.8 ± 5.2 c	27.4 ± 3.8 c	25.6
25	-	62.5 ± 7.0 d	88.0 ± 0.9 d	36.0
30	99.0 ± 1.0 c	92.3 ± 2.2 e	96.2 ± 0.0 e	44.1
40	100.0 ± 0.0 c	100.0 ± 0.0 e	100.0 ± 0.0 e	56.5
50	100.0 ± 0.0 c	100.0 ± 0.0 c	100.0 ± 0.0 e	68.9

Mean values within each column followed by the same letter are not significantly different (*p* < 0.05).

**Table 4:** Probit analysis of the mortality of *P. citri* fumigated with methyl bromide.

Fumigant	Stage	*n*	LCt_50_ (g∙h/m^3^) (95% Conf. Limits)	LCt_99_ (g∙h/m^3^) (99% Conf. Limits)	Slope ± SE	*Df*	χ^2^
Methyl bromide	Egg	812	16.3 (12.8–18.9)	34.6 (31.2–39.5)	7.08 ± 0.73	7	192.3
	Nymph	1006	19.0 (11.5–26.5)	97.7 (55.3–567.2)	3.00 ± 0.53	7	253.2
	Adult	1800	21.1 (8.7–31.0)	86.4 (53.8–388.3)	3.86 ± 0.78	13	783.6

**Table 5:** Probit analysis of the mortality of *T. urticae* exposed to low temperature (1 °C).

		Estimated Days to Achieve % Mortality (LT ^a^, 95% Confidential Limits)		
Stage	Temperature (°C)	Slope ± SE	LT_50_ (day)	LT_99_ (day)
Egg	1.0	1.640 ± 0.334	19.24 (9.43–415.53)	>500
Nymph	1.0	0.731 ± 0.183	36.48 (12.38–463.71)	>1000
Adult	1.0	0.748 ± 0.216	70.31 (16.338–2566.61)	>1000

^a^ Lethal time for 50% and 99% mortality.

**Table 6:** Probit analysis of the mortality of *P. citri* exposed to low temperature (1 °C).

		Estimated Hours to Achieve % Mortality (LT, 95% Confidential Limits)		
Period	Temperature (°C)	Slope ± SE	LT_50_ (h)	LT_99_ (h)
Egg	1.0	3.575 ± 0.786	8.52 (0.008–18.79)	38.17 (17.76–inf)
Nymph	1.0	3.396 ± 0.690	7.30 (0.15–13.37)	35.36 (17.76–6148.09)
Adult	1.0	4.384 ± 0.493	8.45 (5.03–11.38)	28.69 (19.67–70.98)

**Table 7:** Mortality of *T. urticae* exposed to methyl bromide under cold treatment (1 °C).

Concentration (g/m^3^)	Egg	Nymph	Adult	Ct Value (g·h/m^3^)
	Mortlaity ± SE (%)	Mortlaity ± SE (%)	Mortlaity ± SE (%)	
0	0.0 ± 0.0 a	0.0 ± 0.0 a	0.0 ± 0.0 a	-
2	5.1 ± 0.3 b	27.1 ± 4.2 b	26.1 ± 4.0 b	7.8
5	35.1 ± 3.4 c	84.2 ± 4.2 c	46.1 ± 10.7 c	14.9
10	100.0 ± 0.0 d	97.2 ± 5.2 d	83.8 ± 6.9 d	23.4
20	100.0 ± 0.0 d	100.0 ± 0.0 d	96.7 ± 5.6 e	30
25	100.0 ± 0.0 d	100.0 ± 0.0 d	100.0 ± 0.0 f	36.2
30	100.0 ± 0.0 d	100.0 ± 0.0 d	100.0 ± 0.0 f	43.8

Mean values within each column followed by the same letter are not significantly different (*p* < 0.05).

**Table 8:** Probit analysis of *T. urticae* exposed to methyl bromide under cold treatment (1 °C).

Fumigant	Stage	*n*	LCt_50_ (g∙h/m^3^) (95% Conf. Limits)	LCt_99_ (g∙h/m^3^) (95% Conf. Limits)	Slope ± SE	*df*	χ^2^
Methyl bromide	Egg	712	9.82 (7.61–11.6)	22.67 (20.82–24.76)	6.40 ± 0.52	7	152
	Nymph	671	8.96 (5.88–11.46)	29.47 (25.41–36.00)	4.50 ± 0.48	7	116.4
	Adult	680	11.98 (6.63–15.97)	45.47 (33.64–86.53)	4.02 ± 0.63	7	186.2

**Table 9:** Synergistic ratio of methyl bromide and cold treatment for *T. urticae*.

Developmental Stage	Synergistic Ratio ^a^ of LCt_50_	Synergistic Ratio ^b^ of LCt_99_
Egg	0.87	0.75
Nymph	1.75	2.39
Adult	1.11	1.47

^a^ Synergistic ratio (SR) = LCt_50_ of methyl bromide alone/LCt_50_ of combined treatment. ^b^ Synergistic ratio (SR) = LCt_99_ of methyl bromide alone/LCt_99_ of combined treatment.

**Table 10:** Mortality of *P. citri* exposed to low temperature (1 °C) for 1 day after fumigation with methyl bromide.

Concentration (g/m^3^)	Egg	Nymph	Adult	CT Value (g∙h/m^3^)
	Mortality ± SE (%)	Mortality ± SE (%)	Mortality ± SE (%)	
0	0.0 ± 0.0 a	0.8 ± 0.8 a	0.0 ± 0.0 a	
2	100.0 ± 0.0 b	95.3 ± 0.3 b	97.8 ± 0.2 b	3.2
5	100.0 ± 0.0 b	98.6 ± 0.3 c	100.0 ± 0.0 b	7.3
10	100.0 ± 0.0 b	100.0 ± 0.0 c	100.0 ± 0.0 b	15.1
20	100.0 ± 0.0 b	100.0 ± 0.0 c	100.0 ± 0.0 b	21.1
30	100.0 ± 0.0 b	100.0 ± 0.0 c	100.0 ± 0.0 b	45.0
40	100.0 ± 0.0 b	100.0 ± 0.0 c	100.0 ± 0.0 b	60.9

Mean values within each column followed by the same letter are not significantly different (*p* < 0.05).

**Table 11:** Enhancement of the efficacy of methyl bromide against *P. citri* by combined treatment.

Developmental Stage	Estimated Dosage to Achieve 100% Mortality Under Single Treatment (a)	Estimated Dosage to Achieve 100% Mortality When Combined with Cold (b)	a/b
Egg	40.0	2.0	20
Nymph	40.0	10.0	4
Adult	40.0	5.0	8

## Data Availability

The original contributions presented in this study are included in the article. Further inquiries can be directed to the corresponding author.
